# Challenging a “Cushy” Life: Potential Roles of Thermogenesis and Adipose Tissue Adaptations in Delayed Aging of Ames and Snell Dwarf Mice

**DOI:** 10.3390/metabo10050176

**Published:** 2020-04-29

**Authors:** Teresa G. Valencak, Tanja Spenlingwimmer, Ricarda Nimphy, Isabel Reinisch, Jessica M. Hoffman, Andreas Prokesch

**Affiliations:** 1 College of Animal Sciences, Zhejiang University, Zijingang Campus, 866 Yuhangtang Road, Hangzhou 310058, China; 2 Department of Biomedical Sciences, Institute of Physiology, Pathophysiology and Biophysics, University of Veterinary Medicine, Veterinärplatz 1, A-1210 Vienna, Austria; 1607141@students.vetmeduni.ac.at (R.N.); 3 Division of Cell Biology, Histology and Embryology, Gottfried Schatz Research Center for Cell Signaling, Metabolism and Aging, Medical University of Graz, Neue Stiftingtalstrasse 6, 8010 Graz, Austria; isabel.reinisch@medunigraz.at (I.R.); andreas.prokesch@medunigraz.at (A.P.); 4 Department of Biology, University of Alabama at Birmingham, 1300 University Blvd., CH464, Birmingham, AL 35294, USA; jmhoffm@uab.edu; 5 BioTechMed-Graz, Mozartgasse 12/II, 8010 Graz, Austria

**Keywords:** white adipose tissue, brown adipose tissue, lipids, IGF-1, Prophet of Pituitary 1 (Prop-I), Pituitary specific factor 1 (Pit-I)

## Abstract

Laboratory mouse models with genetically altered growth hormone (GH) signaling and subsequent endocrine disruptions, have longer lifespans than control littermates. As such, these mice are commonly examined to determine the role of the somatotropic axis as it relates to healthspan and longevity in mammals. The two most prominent mouse mutants in this context are the genetically dwarf Ames and Snell models which have been studied extensively for over two decades. However, it has only been proposed recently that both white and brown adipose tissue depots may contribute to their delayed aging. Here we review the current state of the field and supplement it with recent data from our labs.

## 1. Introduction—Aging and the Somatotropic Axis: A Focus on Ames and Snell Mice

It is well accepted in the biology of aging that insulin-like growth factor 1 (IGF-1) and insulin signaling relate to longevity in an evolutionarily conserved manner [1,2]. Reduced IGF-1 and insulin signaling significantly extends longevity in all model systems in which it has been tested including yeast (*Saccharomyces cerevisiae*), worms (*Caenorhabditis elegans*), flies (*Drosophila melanogaster*), and mice (*Mus musculus*) (reviewed in [3]). Downregulation of growth hormone (GH), acting upstream of IGF-I, extends lifespan in mammalian models (review in [4]). While GH is secreted from the pituitary, IGF-1 as well as its auxiliary hormones, proteins, and receptors are synthesized in the liver and together these components form the pituitary-somatotropic axis [1,4,5,6].

To this end, many mutations that affect the somatotropic axis have been extensively explored in mice to understand the functional relationships between GH, IGF-I, and lifespan (reviewed in [1,4,7]). Among the described long-lived GH mutants, the Ames dwarf (AD) and Snell dwarf (SD) mice have received the most attention because they were natural mutations that were discovered early on with delineation of the causative loss of function mutation and the direct effects on healthspan and lifespan ([8]; summarized in [9,10]).

AD and SD mice are born normal sized but then show retarded growth from ~10 days of age which is clearly recognizable even well before weaning. In addition to stunted growth, AD and SD mice have a number of phenotypical similarities that are summarized in Table 1 and mentioned throughout the text. 

In humans, there is no consistent evidence for longevity extension in individuals with mutations related to GH signaling [4]. Rather, studies in humans with various hereditary dwarfing syndromes (including the mutations causal for AD and SD phenotypes, see below) produced conflicting findings with examples of reduced, unaltered, or possibly extended longevity, summarized in [4]. Notably in humans, mutations affecting the phenotype are also seen in compound heterozygous individuals, not only in homozygous individuals as in AD [4]. 

## 2. AD and SD Mice as Models for Delayed Aging

### 2.1. AD Mice—Old and New Observations about Endocrine and Metabolic Disruptions, Outward Phenotypes, and Aging

The gross phenotype of AD mice (see Table 1 for hallmark changes) is caused by a point mutation in the recessive Prophet of Pituitary 1 (Prop 1) gene which results in a failure of differentiation of endocrine cells in the pituitary (summarized in [20,25]). Thus, AD mice are observed to have an irreversible endocrine disruption with the lack of somatroph, thyrotroph, and lactotroph cells in the pituitary (reviewed in [4]) and are consequentially void of somatotropin, thyroid-stimulating hormone (TSH), and prolactin (PRL) [26]. However, other pituitary hormones such as the gonadotropins, follicle stimulating hormone (FSH) and luteinizing hormone (LH), are lower but still detectable [27]. Equally, another important pituitary hormone, the function of the adrenocorticotropic hormone (ACTH) is conserved in AD mice and its blood serum levels are increased in response to stress similarly to controls [12]. As for the seventh pituitary hormone, the anorexigenic peptide melanocyte stimulating hormone (MSH), it was also found to be reduced compared to heterozygous controls [13]. Interestingly, if AD mice were given GH injections early in life, they had MSH function comparable to heterozygous littermates [13]. In AD mice, this broad endocrine disruption results in reduced body size to approximately 10 g when mature, lower body temperature of about 34.9 °C [28], a lower respiratory quotient [14], lower IGF-1 and insulin levels [4] and suppressed serum glucose [9] when compared to littermate controls. 

These physiological changes all contribute to metabolic differences in AD compared to controls. We reported previously that AD mice have significantly lower n-3 polyunsaturated fatty acids as measured in several tissues, probably relating to oxidative damage, as discussed in 3.3 [19]. However, the metabolic rate is not simply slowed down as would be predicted from low TSH. Rather, indirect calorimetry measurements indicated increased oxygen consumption (VO_2_) per gram of bodyweight (and even more so per unit of lean body mass) and a decreased respiratory quotient in both ad libitum fed and fasted animals [1]. This increased metabolic rate in AD mice was unexpected and interpreted as a consequence of increased energy expenditure for thermogenesis. AD mice have elevated heat radiation due to an increased body surface to mass ratio in these diminutive animals [1]. Reductions in production of reactive oxygen species (ROS) in skeletal muscle, suggesting increased mitochondrial efficiency, have also been reported [17]. 

AD mice of both sexes live longer than heterozygous siblings [8] and their mean lifespan was reported as 718 ± 45 d for AD males and 1076 ± 56 days for females, respectively [8]. According to the “disposable soma theory of aging” [29], the extended lifespan in AD and SD is commonly attributed to the reduced body size. More specifically, the deficiency of TSH renders AD mice hypothyroid which again plays into the “disposable soma theory” [8,29]. In addition to the mild hypothyroidism, the lower insulin signaling is proposed to lead to a youthful phenotype with an intact immune system until late life ([8]; reviewed in [20]). The lack of prolactin causes infertility in females, while males reportedly can breed normally [30] but are considered subfertile, with the degree of gonadal function being dependent upon the genetic background [21,30]. Yet, hypothyroidism and hypogonadism prevent the mutants from excessive energy expenditure for reproduction as common in female rodents. In addition, recent reports suggest that other markers of aging are improved with lower cartilage necrosis and thus lower osteoarthritis severity in AD mice [31] as well as lower levels of “inflammaging”, i.e., chronic, low-grade sterile inflammation that occurs during aging [32]. In addition, gut microbiomes of AD mice are altered compared to their heterozygous controls, with higher levels of *Muribaculaceae* and lower levels of *Rikenellacae* suggesting an overall more juvenile microbiome composition [33].

### 2.2. SD Mice—Similar but Still Not Alike?

SD mice, named after George Snell in 1929 [34], are long-lived when homozygous for a point mutation in the pituitary- specific factor 1 (*Pit 1*) gene. During embryonic development, *Prop 1* acts as transcription factor that regulates the expression of *Pit 1* (reviewed in [4]; thus, many phenotypes are shared between animals with *Prop 1* and *Pit 1* mutations. The *Pit 1* gene product is a transacting POU domain protein, also called class 1 transcription factor 1 gene (*Pou1f1*), and is necessary for the production of somatotrophs, thyrotrophs, and lactotrophs in the anterior pituitary (reviewed in [4]. Therefore, SD mice were found to have lower GH, IGF-1, TSH, thyroid hormones (T3 and T4), and PRL, as well as pituitary hypoplasia [4,26]. The mutation in the *Pit1* gene further leads to hypopituitarism [4]. Contrary to AD, SD mice were reported to have reduced metabolic rates (i.e., lower VO_2_ than controls [15] as would be expected from the low TSH and thyroid hormone levels (Table 1). 

**Figure 1:**
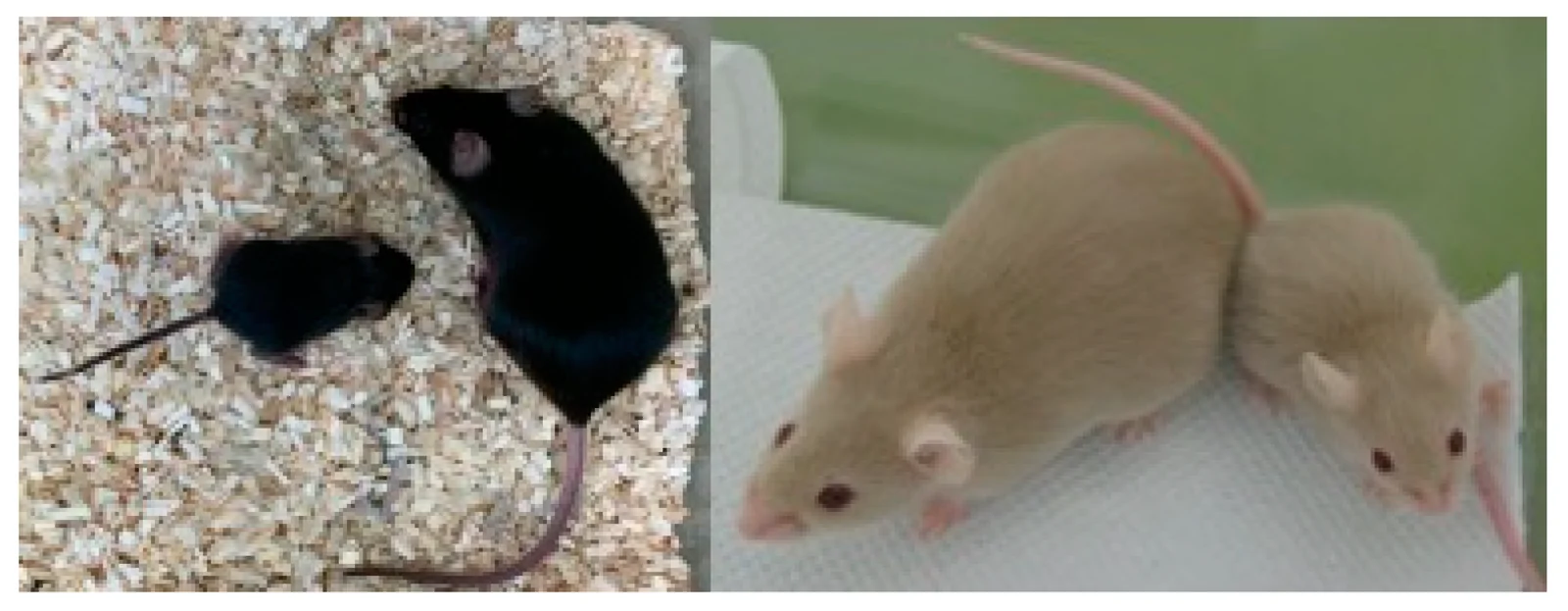
Homozygous SD and heterozygous sibling mouse (left panel) and heterozygous control with AD sibling (right panel). Picture by T.G. Valencak and S.A. Ohrnberger.

Similar to AD, SD mice reach only one third of the adult size of their heterozygous siblings (Figure 1) [26] and their lifespan is extended 40–50% [35]. Mean lifespans have been reported previously at 618 ± 87 days in SD males and 844 ± 44 days in SD females, respectively. Initial studies reported some immunological impairments indicating some T-cell dependent functional loss [36] and suggested SD as a model for accelerated senescence [36,37]. The T-cell dysfunction was later disproven by showing consistently that SD outlive control individuals [35], particularly when special attention was paid to ideal husbandry (see below). Importantly, if male SD and male control littermates were co-housed together in one cage, lifespan was observed to be lower. The above mentioned 40–50% lifespan extension manifested only when males were maintained together with female (not male) control “caretakers” [35].

### 2.3. Lower Body Temperature in SD Mice

To our knowledge, no study has assessed core body temperature in SD mice so far. So, we subcutaneously implanted passive integrated transponders (PIT) tags, as successfully used and described recently in Lenzhofer et al. [11]. After the temperature-sensitive transponders were safely implanted, we measured the subcutaneous temperatures daily at the same time for 14 days in both SD and heterozygous control animals. Surprisingly, the temperature difference was almost 4 °C between the two groups: Homozygous SD mice had mean subcutaneous temperatures of 32.43 ± 0.3 °C vs. 36.4 ± 0.3 °C in the control. SD mice thus had an even 2 °C lower subcutaneous temperature than AD mice (see above), an overall lean phenotype, and lack of a thick body integument (Figure 1).

Thus, we suggest the most obvious explanation for the low skin temperature in SD mice is that they were torpid when being measured daily (at the same time; 9–10 a.m. on a 12:12: L:D with lights on at 6 a.m.). Entering a temporary state of torpor to compensate for low energy intake is well known in mice [38] and ecologically is linked to predator avoidance [38,39]. Also, core body temperatures in torpid house mice were reported to be 31.32 °C ± 3.76 [39] or, even as low as 24.8 °C [39] SD mice may therefore drastically lower their daily energy expenditure through being in a torpor-like state throughout most of their inactive phase with the consequence of being less alert and reinforcing the low foraging effort for food (see below). Our so far anecdotal observation that SD are more likely to go into torpor than AD will require more research in the future.

### 2.4. Husbandry and Feeding Behavior of AD and SD Mice

**Figure 2:**
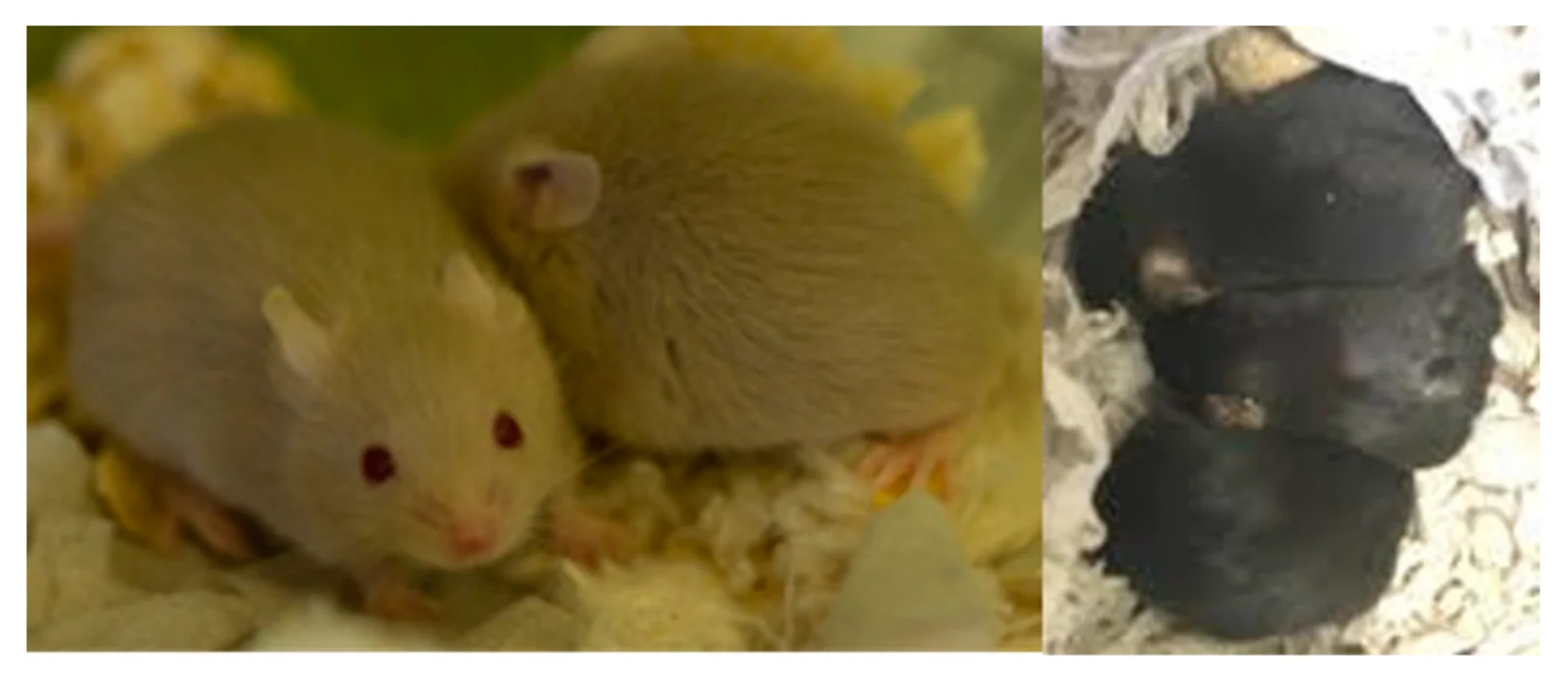
Social thermoregulation in AD (left panel) and SD mice (right panel). Pictures by K. and S.A. Ohrnberger.

Implementing ideal husbandry conditions for AD and SD mice has taken decades with the common theory that they should be housed together with normal sized control littermates to ensure maintenance of their normothermic body temperature [8,11,35]. Equally, separate housing of control and AD mice is possible, but individual housing of AD and SD mice is not ideal. From a physiological point of view, the co-housing of SD with normal sized controls enables them to benefit from “social thermoregulation” which is also known as “huddling” and commonly performed across mammalian species [40]. The social regulation of body temperature helps animals to warm each other during times of danger, disease or distress and importantly also, during torpor and hibernation [41]. This very common behavioral strategy to conserve heat in endothermic mammals may be particularly relevant for genetically dwarf mice such as AD and SD and indeed we observed this behavior broadly in our AD and SD colonies (Figure 2). From literature on hibernators such as marmots it is known that juveniles, having the lowest body fat reserves, benefit most from huddling as it significantly decreases their energy costs for endogenous thermoregulation [41]. Thus, we derive from our observations, that in addition to the importance of co-housing AD or SD mice with female caretakers, it is also imperative to provide them with adequate nest material to facilitate huddling together. In SD colonies, these nests preferentially should be located in the vicinity of food pellets to restrict the necessity for foraging efforts when resting and huddling in the nest (Figure 2). 

After having successfully bred and raised AD mice for almost 10 years, our laboratory noticed several differences when trying to extrapolate our experiences to SD mice. In contrast to AD mice that thrive on a normal chow diet, we discovered that SD mice require a calorically enriched diet (such as mouse breeding chow extra enriched diet V1185-000 from Ssniff, Soest, Germany, gross energy 17.2 MJ/kg) to successfully grow and develop. We observed that while they survive normally on the conventional breeding chow, growth rates of young and breeding success of the females was lower. Further, warmer ambient housing temperatures of 24 ± 2 °C are preferential, although even under these improved conditions SD mice still were less active, had lower body mass, and were less alert than AD mice in the same age class. Surprisingly, despite the high energy content of their breeding diet, both SD phenotypes (dwarf and controls) were also leaner and lighter than AD mice and AD controls from the same age cohort. However, due to the co-housing with the normal-sized “caretaker” mice, it was impossible for us to receive accurate individual food intakes in this setup.

In summary, husbandry of AD and SD mice has different demands compared to normal sized laboratory mice and special attention has to be given to co-housing and nesting material to fulfill their thermoregulatory needs. While AD mice have been successfully maintained in a conventional laboratory environment [8,11,19], SD mice have to be kept in filter-hooded cages in a specific pathogen-free environment and need chlorinated drinking water acidified to prevent the growth of Pseudomonas [35], all of which is increasingly becoming today’s standard maintenance conditions of laboratory rodents.

## 3. Specifics of Adipose Tissue Depots in AD and SD Mice

### 3.1. Adipose Tissue—Communalities, Differences, and Function

Along with the above described alterations in their metabolism, genetically dwarf mice were found to have functionally altered adipose tissues (reviewed in [6]). Generally, three types of adipose tissue are found in mammals: white, brown, and beige. White adipose tissue (WAT) is considered the body’s energy storage for times of energy scarcity while brown adipose tissue (BAT) is a unique, major energy consuming, heat producing organ. This highly thermogenic BAT, found commonly in small sized mammals and juveniles of larger-bodied mammals including humans, is very important for physiology in general [42,43] and metabolic homeostasis in particular [44]. It not only maintains endothermy but also is crucial for many physiological processes relating to decreased metabolic rate i.e., hypometabolism, daily torpor, and the longest and deepest torpor lasting up to several months (i.e., hibernation; reviewed in [45]). Lastly, beige or brite adipose is originally derived from WAT precursors but has properties more similar to BAT [46,47].

For decades, both WAT and BAT were largely excluded from evolutionary and developmental research in cell and tissue biology. Due to the common notion that adipose tissue was mainly assigned a passive role for lipid storage, insulation and mechanical buffering it was considered a large source of unwanted biological variance due to individual feeding status and other environmental factors driving the extent and composition of WAT and BAT. More recently, WAT has been recognized as a major endocrine organ, and as such, the interest in adipose tissues has increased dramatically. The various functions of WAT have been examined to be seasonally regulated and even involved in complex physiological processes such as immune responses in both wild and laboratory animals (reviewed in [48]). Finally, adipocytes in the mammary fat pad can give rise to epithelial cells producing fat-rich milk in the mammary gland. These adipocytes of white origin are commonly referred to as pink adipocytes [49,50].

**Figure 3:**
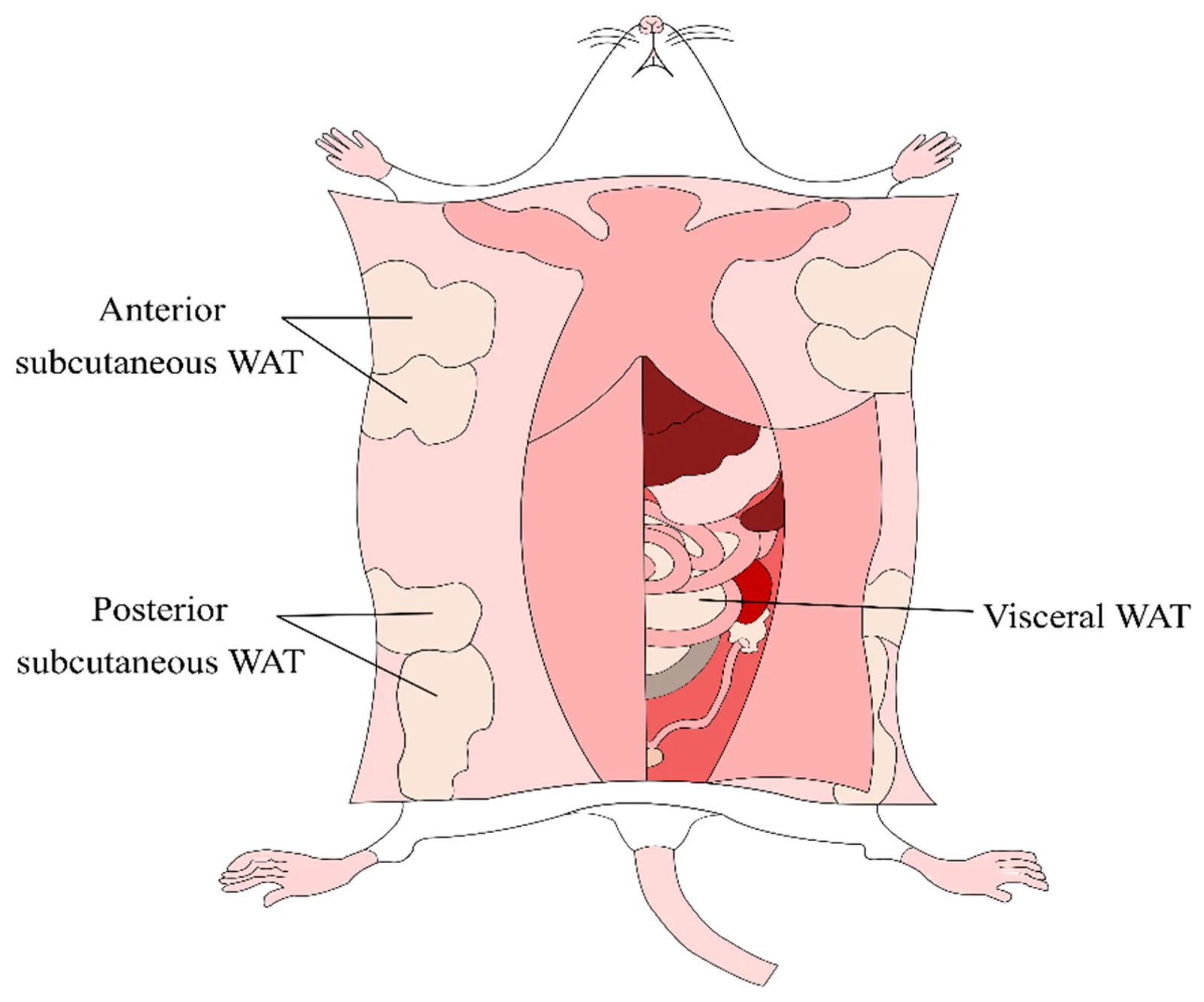
Localization of the three, most relevant adipose tissue depots for AD and SD mice. Scheme by T. Spenlingwimmer. WAT: White adipose tissue.

WAT is generally found in all vertebrates, but the localization and functional regulation is species-specific, while the secretion of adipokines is a generally conserved function [48,51]. Extant fish species store triacylglycerols (TGs) mostly in the liver and/or skeletal muscle [52], and in amphibians, stored TGs molecules are found as fat bodies in the abdomen [48,53]. Reptiles store TGs in paired abdominal fat bodies, in adipose tissue depots in the tail, and in the abdominal cavity with poorly developed subcutaneous WAT (reviewed in [54]). Adipose depots in mammals have been best studied in rodents with at least twelve different locations, most of which are thermo-active as shown by glucose and fatty acid uptake upon cold exposure, and have human equivalents visualized in PET/CT scans [55,56]. Figure 3 depicts the three most important fat pads for AD and SD described in more detail in our present paper and thus only provide a selection of fat pads generally observed in mice [55]. Notably, many of these fat pads have site-specific functions for thermoregulation, structural distinctions [56], or paracrine interactions with other tissues.

### 3.2. Expandability of Subcutaneous WAT in AD Mice: Is There a Beneficial Role for Overall Energy Metabolism?

Interestingly, there seem to be peculiarities in WAT localization in homozygous long-lived AD mice compared to normal sized, heterozygous controls. The potential differences in WAT depots compared to other laboratory mice became most visible when AD were exposed to a high fat diet containing 60% fat [57]. Diet-induced obesity in AD seemingly did not lead to expected metabolic derangements which clearly developed in littermate controls, despite significant increases in the amount of their subcutaneous and visceral depots [57]. Instead, “obese” AD mice remained insulin sensitive and showed normal levels of adiponectin [57]. The adipokine adiponectin, acts as an important anti-inflammatory factor and usually correlates positively with the retention of insulin sensitivity [58].

In contrast to control mice which showed improvements in insulin signaling upon surgical removal of visceral WAT for glucose turnover and insulin action, AD mice undergoing visceral fat removal did not have improved glucose tolerance in skeletal muscle but did have decreased blood glucose levels [59]. This finding suggests that not only does visceral WAT play a positive role in maintaining whole-body insulin sensitivity in AD [59], but we can even speculate that adequate visceral WAT depots are required for glucose management and insulin action in AD. Potentially, expandability of visceral WAT may even be considered metabolically beneficial [60].

Similarly, by manipulating the fatty acid composition in the diet, we observed that AD mice readily increased body mass by 23% (compared to 16.7% in the controls) [11]. While the origin of fat was either saturated, n-3 or n-6 fatty acids, we observed no differences in body weight gain relating to fatty acid origin [11]. Otherwise, AD showed no signs of adverse health effects after 6 weeks on isocaloric high fat diets differing only in fatty acid composition [11]. Interestingly, we measured a significant increase in subcutaneous body temperature in AD mice (0.45 °C) following the exposure of fatty acid-enriched diets (saturated, n-3 and n-6 enriched) which was not present in controls [11]. More specifically, we observed that the AD mice on the fatty acid-enriched diets had increased subcutaneous fat mass as compared to controls (similar to what was observed in [11]). This surplus subcutaneous WAT clearly improved body insulation as mice became more active in their behavior [11]. As discussed above, genetically dwarf mice such as AD and SD are challenged in their thermoregulation due to their lower body temperature and their disadvantageous surface area to volume ratio. Hence, an increase in volume of the subcutaneous WAT layer may be particularly advantageous for their overall energy budget. Being able to save on thermoregulatory energy costs, genetically dwarf mice may be able to allocate energy into other avenues such as a decreased time spent in torpor or increased foraging and general activity. 

We thus hypothesize here that GH-deficient, genetically dwarf mice, such as AD and SD, have a metabolic advantage when kept on high-fat diets through the storing of triglycerides (TGs) preferentially in subcutaneous depots as opposed to evoking depots around the visceral organs like many common laboratory mouse models. This is important as visceral WAT is primarily associated with metabolic complications such as insulin resistance, increased inflammation and even cancer, which have detrimental effects on tissue health and metabolism [61,62]. To date, no adverse metabolic effects are described from expansion of subcutaneous WAT. Rather subcutaneous WAT has been assigned metabolic beneficial roles through its browning ability [63]. An alternative explanation could be an overall increased capacity of genetically dwarf mice to expand fat depots when fed high-fat diets, a mechanism that was suggested to provide health benefits in obese animal models and individuals [64,65]. The hypothesis we put forward here on the potential beneficial effects of subcutaneous WAT in dwarf mice was derived from observing AD mice exposed to diets enriched with saturated, n-6 and n-3 polyunsaturated fatty acids [11] and future studies in SD mice, exposed to respective fat-enriched diets differing in fatty acid composition, will have to address specifics of subcutaneous WAT remodeling in this model. 

### 3.3. Non-Shivering Thermogenesis in GH-Deficient AD and SD Mice

Undoubtedly, the capability of both AD and SD mice to conserve euthermia via non-shivering thermogenesis (i.e., the capacity of an endothermic mammal to uncouple respiration from ATP production in BAT mitochondria and thereby producing heat) is key to their survival. In particular, the vulnerability for thermoregulation in AD and SD mice may arise from their larger body surface to body mass ratio, suggesting that they are subjected to a higher thermal loss through their skin [1]. Indeed, increased weight of BAT was observed in AD mice although it showed reduced cell size and size of lipid droplets. Not surprisingly, the key enzyme triggering uncoupled respiration and consequent heat production in BAT mitochondria, uncoupled protein 1 (Ucp1), was found to be largely upregulated in AD mice [66,67]. and that Ucp1 expression correlated negatively with GH signaling in interscapular BAT (iBAT) [68]. Similarly, the gene expression of two transcriptional regulators in the iBAT, PPARγ, and PPARγ coactivator 1α (PGC1α), were significantly increased in the AD mice [66]. Altogether, contrary to their lower body temperature, AD mice show increased thermogenic marker expression in iBAT and a direct mechanistical link with their endocrine disruptions has not yet been investigated. 

**Figure 4:**
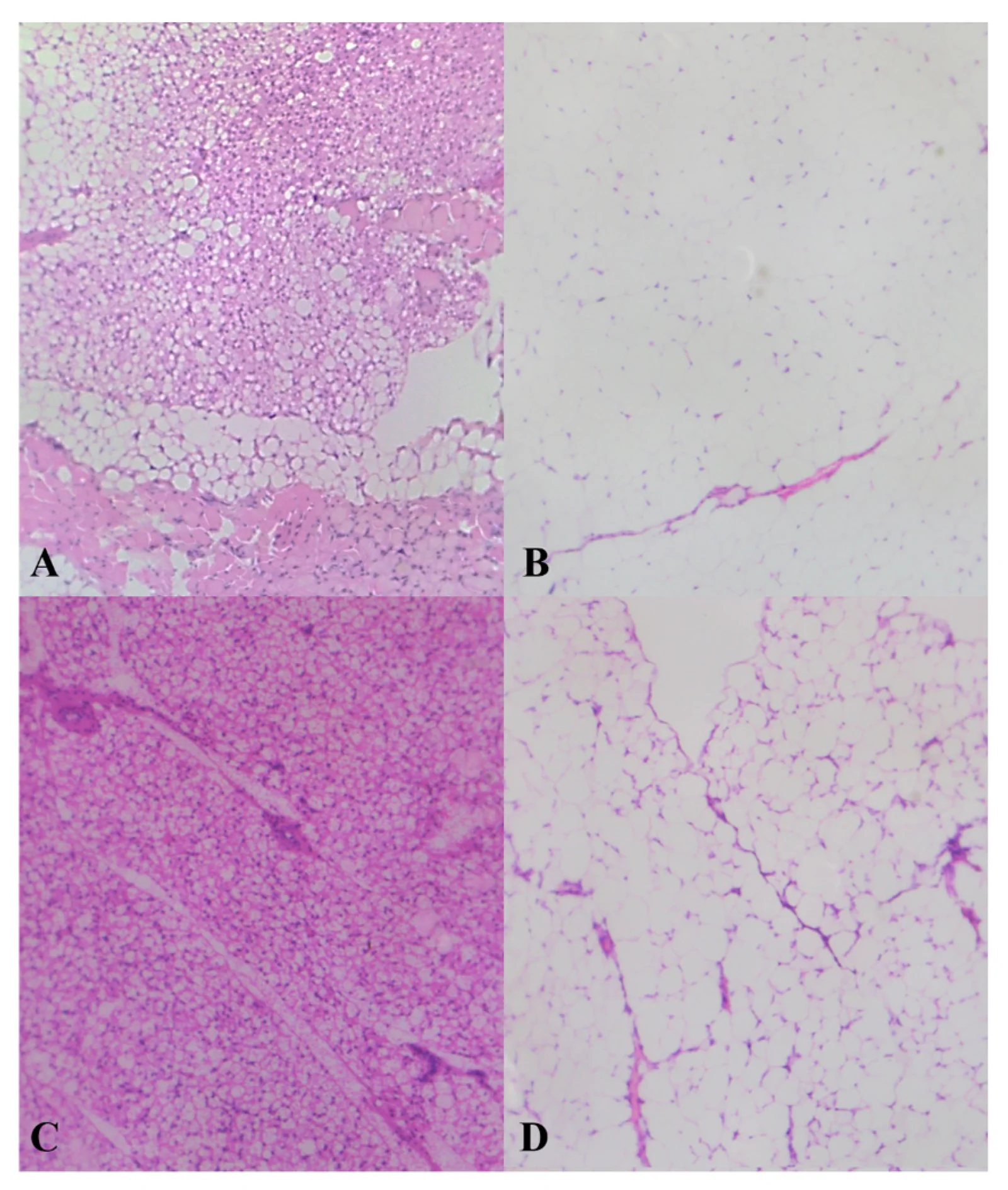
HE staining of interscapular BAT (iBAT) (**A**,**C**) and visceral WAT (**B**,**D**) of a homozygous SD and a heterozygous control respectively. Pictures by T.G. Valencak and K. Brugger.

In light of the above facts from AD mice, we set out to inspect iBAT and Ucp1 expression in SD mice which, to our knowledge, has not yet been addressed. As can be seen from Figure 4, iBAT was visually recognizable and confirmed by the classical histological appearance of small multi-locular adipocytes. Notably, Figure 4 represents a first, pilot comparison of WAT and BAT from homozygous SD and controls while future research will have to quantify the thermogenic capacity of SD by norepinephrine induction. 

**Figure 5:**
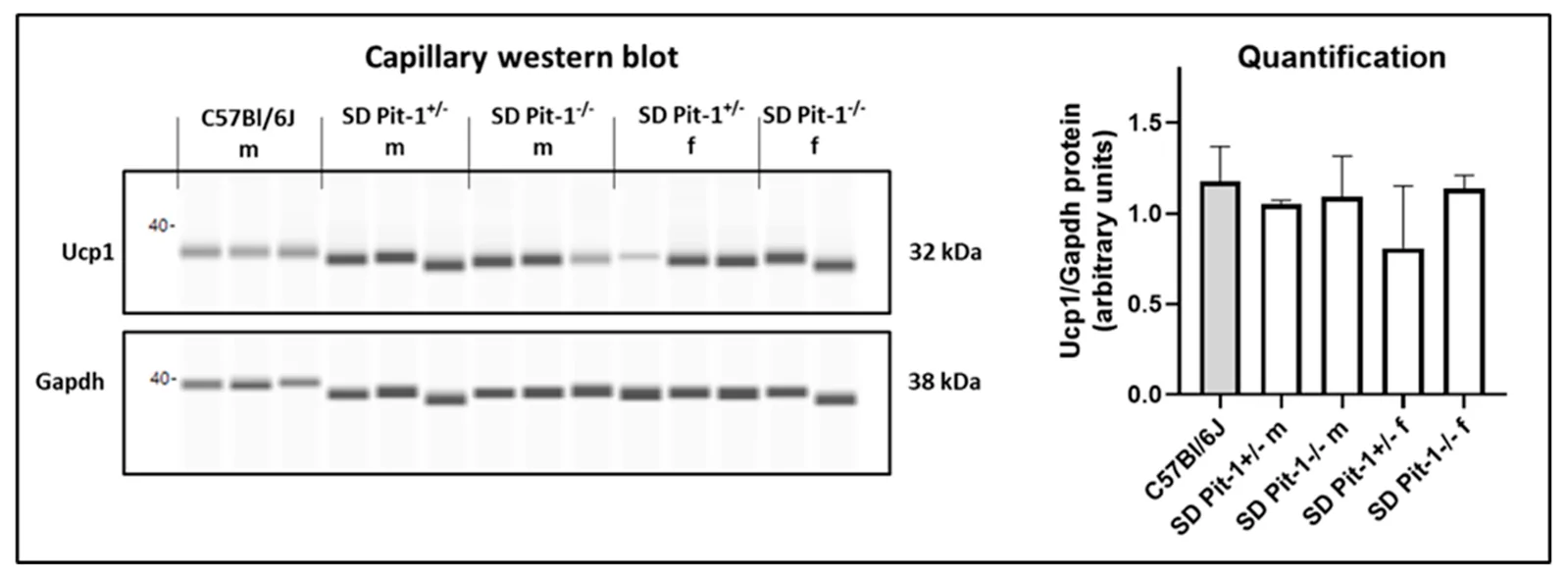
Capillary western blot (ProteinSimple, Bio-Techne) results BAT protein lysates from male (m) or female (f) SD mice and heterozygous controls, showing Ucp1 protein (ab 10983, Abcam, Cambridge, UK) and Gapdh as loading control. BAT samples from standard C57Bl/6J mice are shown for comparison. Right panel shows quantification of electropherogram peaks (AUC) according to blot in the left panel.

When comparing Ucp1 protein levels between iBAT from SD mice and C57Bl/6J mice (Figure 5) to assess functionality of iBAT in SD mice, we detected similar levels upon normalization to loading control (Figure 5, right panel). Furthermore, we could not detect any significant differences in Ucp1 expression between SD and heterozygous controls (Figure 5). Therefore, we can confirm the histological picture (Figure 4) that SD mice possess iBAT but Ucp1 protein levels were not different from normal-sized control mice (Figure 5). We are well aware that mere expression of Ucp1 is not a faithful determinant of thermogenic activity of iBAT [69]. Equally, the impact of the genetic background (C57Bl/6) on BAT histology requires testing before robust comparisons with AD from a heterogenous outbred background can be done. Hence, further studies should be undertaken to clarify why (i) SD mice survive better in warm ambient temperatures, (ii) SD mice use torpor to reduce and manage their energy expenditure, and (iii) the *Pit1* mutation does not equally affect non-shivering thermogenesis in SD mice, as the *Prop1* mutation does in AD mice.

### 3.4. A Role for Polyunsaturated Fatty Acids in Adipose Tissue of AD and SD Mice?

Polyunsaturated fatty acids (PUFAs) are essential membrane constituents, precursors for eicosanoids, functionally affect complex cellular processes such as immune responses and reproduction [51], and act as signaling molecules, for instance as agonists for the pro-adipogenic transcription factor peroxisomal proliferator-activated receptor gamma (PPARγ) [70]. All mammals must obtain linoleic and alpha-linolenic acid from their diet to enzymatically synthesize longer chained PUFAs. Dietary supply of PUFAs therefore plays a large role in the expansion of WAT depots as well as their lipidomic profiles. Interestingly, membrane fatty acid composition is a tightly regulated physiological trait in mammals where membranes of small mammals are rich in n-3 PUFAs and larger bodied mammals have predominantly n-6 fatty acids [71,72]. It has been suggested within the framework of the “membrane pacemaker hypothesis of aging” that higher levels of membrane n-3 PUFAs, specifically the long-chain docosahexaenoic acid C 22:6 n-3, may give rise to oxidative stress and thereby may explain the shorter life in mice in comparison to much larger mammals such as ungulates [73,74]. Previously, we observed in AD mice that membrane-bound, n-3 PUFAs were indeed lower than in the controls [71]. When exposed to a diet enriched with n-3 PUFAs, AD doubled the heart phospholipid n-3 content without any visible adverse effects and leaving many open questions. Conversely, a diet enriched with n-6 PUFAs increased heart phospholipid n-6 fatty acids by 2% [11]. While the effects of these changes in membrane composition on membrane fluidity, inflammatory cytokines and other involved pathways still remain to be elucidated, the uncoupling function of PUFAs on membrane bound proteins, specifically UCP-1, is well known [43,45] and largely influences thermogenesis in mammals.

Dietary and membrane PUFAs also play an important role in regulating metabolism during torpor and hibernation (reviewed in [45,75]. Specifically, high dietary PUFAs or PUFAs in WAT stores have a positive effect on the propensity of animals to enter torpor, on the duration of torpor bouts on minimum body temperatures tolerated, and on energy reserves [45]. Involved transmembrane proteins and pathways by which PUFAs and monounsaturated fatty acids exert their influence on hibernation are still under scrutiny [45]. Thus, we hypothesize that the role of PUFAs in energy metabolism of AD as well as SD mice are particularly relevant due to their lower body temperature (Table 1, [11,28]). We speculate that the altered n-3 to n-6 ratio as previously observed in AD (Table 1, [19]) may resemble membrane compositions of hibernators at the onset of winter [42]. Also, the frequent use of torpor throughout day and night in AD and SD should become an important subject of further investigation. Closely connected to the relationship between body temperature and torpor are active food foraging efforts and food intake, which seems to be higher in AD than in SD mice. Future studies in genetically dwarf mouse models should therefore involve dietary supplementation with either n-6 or n-3 fatty acids. Providing them with enriched diets may thus help to unravel the functional relationships between PUFAs, membrane composition, body temperature, and lifespan.

## 4. Concluding Remarks and Future Directions

Here, we reviewed the existing literature on AD and SD mice by focusing on the understudied role of subcutaneous WAT. Subcutaneous WAT effectively conserves body heat in these small animal models in which maximal heat dissipation and loss through their body surface with co-existent lower body temperature exists. We also elaborated on specific husbandry requirements in genetically dwarf mice and initiated first evaluations of BAT function in SD mice. Overall, we suggest that future experimental studies in SD mice should involve diets enriched in n-3 or n-6 PUFAs with a special focus on remodeling of, and altered functions in, subcutaneous WAT and iBAT. Thus, it is conceivable that the SD model strives to increase its food and energy intake on the enriched diets to establish an insulating subcutaneous WAT layer, enabling them to reduce the total time spent in energy-saving torpor and rather stay euthermic, active, and foraging. We consider the relationships between the somatotropic axis, adipose tissue function, and body temperature as eminently important to better understand the “vigor of survival” in the long-lived AD and SD mice [35]. 

## Figures and Tables

**Table 1:** Overview of phenotypic properties reported from Ames dwarf (AD) and Snell dwarf (SD) mice. Decreases and increases are always compared to heterozygous controls. For those traits being measured by several laboratories we cite averages calculated from previous reviews. Please note that the size of the arrow indicates the magnitude of change.

	AD 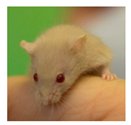	Reference	SD 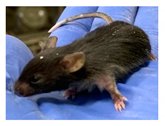	Reference
Body mass (g)	13.53 ± 0.5	[11]	9.6 ± 1.2	this study
Subcutaneous temperature (°C)	34.5 ± 0.5	[11]	32.43 ± 0.3	this study
Growth Hormone (GH)	↓	[1,4,5,6]	↓	[1,4,5,6]
Insulin-like growth factor1 (IGF-1)	↓	[1,4,5,6]	↓	[1,4,5,6]
Thyroid-stimulating hormone (TSH)	↓	[1,4,5,6]	↓	[1,4,5,6]
Follicle-stimulating hormone (FSH)	↓	[6]	↓	[6]
Luteinizing hormone (LH)	↓	[6]	↓	[6]
Prolactin (PRL)	↓	[6]	↓	[6]
Adrenocorticotropic hormone (ACTH)	unchanged	[12]	NA	
Melanocyte-stimulating hormone (MSH)	↓	[13]	NA	
Metabolic rate (heat per gram body weight in calories per hour)	↑ (AD 336.4 ± 11.7 vs. 279 ± 7.3 in Controls)	[14]	↓	[15]
Oxygen consumption (VO_2_)	↑ (AD 55 mL/kg/min vs. 43 in Controls)	[14]	NA	
Respiratory Quotient (fasted animals)	↓ (AD 0.7 vs. 0.76 in Controls)		NA	
Mitochondrial stress response	NA		↑	[16]
Reactive oxygen species (ROS)	↓	[17]	↓	[18]
Triiodothyronine (T3), Thyroxine (T4)	↓ (below detection limit)	[1,4,5,6]	↓	[1,4,5,6]
Heart phospholipid n-3 Polyunsaturated fatty acids (n-3 PUFAs)	25.6 ± 1.3 AD vs 35.3 ± 0.7 Controls	[19]	NA	
Maximum lifespan (MSLP) [days]	1206 ± 32 females, 1076 ± 56 males	[20]	1148 ± 39 females1037 ± 53 males	[20]
Onset of puberty	delayed	[21,22]	delayed	[21,23]
Female Fertility	Absent	[21]	Absent	[21]
Male Fertility	subfertile	[22]	subfertile	[22]
Uncoupling protein-1 (UCP-1) mRNA expression	↑	[20,24]	yes	this study
